# Molecular identification and anti-malarial drug resistance profile of *Plasmodium falciparum* from patients attending Kisoro Hospital, southwestern Uganda

**DOI:** 10.1186/s12936-021-04023-3

**Published:** 2022-01-15

**Authors:** Godfrey Manirakiza, Kennedy Kassaza, Ivan Mugisha Taremwa, Joel Bazira, Fredrick Byarugaba

**Affiliations:** 1 Department of Microbiology, Faculty of Medicine, Mbarara University of Science and Technology, Mbarara, Uganda; 2 Institute of Allied Health Sciences, Clarke International University, Kampala, Uganda

**Keywords:** Dried blood spots, High-resolution melting analysis, *Plasmodium species*

## Abstract

**Background:**

The evolution of malaria infection has necessitated the development of highly sensitive diagnostic assays, as well as the use of dried blood spots (DBS) as a potential source of deoxyribonucleic acid (DNA) yield for polymerase chain reaction (PCR) assays. This study identified the different *Plasmodium* species in malaria-positive patients, and the anti-malarial drug resistance profile for *Plasmodium falciparum* using DBS samples collected from patients attending Kisoro Hospital in Kisoro district, Southwestern Uganda.

**Methods:**

The blood samples were prospectively collected from patients diagnosed with malaria to make DBS, which were then used to extract DNA for real-time PCR and high-resolution melting (HRM) analysis. *Plasmodium* species were identified by comparing the control and test samples using HRM-PCR derivative curves. *Plasmodium falciparum* chloroquine (CQ) resistance transporter (*pfcrt*) and *kelch13* to screen the samples for anti-malarial resistance markers. The HRM-PCR derivative curve was used to present a summary distribution of the different *Plasmodium species* as well as the anti-malarial drug profile.

**Results:**

Of the 152 participants sampled, 98 (64.5%) were females. The average age of the participants was 34.9 years (range: 2 months–81 years). There were 134 samples that showed PCR amplification, confirming the species as *Plasmodium*. *Plasmodium falciparum* (N = 122), *Plasmodium malariae* (N = 6), *Plasmodium ovale* (N = 4), and *Plasmodium vivax* (N = 2) were the various *Plasmodium* species and their proportions. The results showed that 87 (71.3%) of the samples were sensitive strains/wild type (CVMNK), 4 (3.3%) were resistant haplotypes (SVMNT), and 31 (25.4%) were resistant haplotypes (CVIET). Kelch13 C580Y mutation was not detected.

**Conclusion:**

The community served by Kisoro hospital has a high *Plasmodium* species burden, according to this study. *Plasmodium falciparum* was the dominant species, and it has shown that resistance to chloroquine is decreasing in the region. Based on this, molecular identification of *Plasmodium* species is critical for better clinical management. Besides, DBS is an appropriate medium for DNA preservation and storage for future epidemiological studies.

## Background

Malaria is still a highly contagious infectious disease that affects half of the world's population [1]. Globally, there are an estimated 229 million malaria cases in 2019 in 87 malaria endemic countries with about 409,000 malaria deaths. The total percentage of malaria deaths among children under 5 years was 67% in 2019 [2]. The burden of malaria is higher among children and pregnant women, but people of all ages are at risk of infection [3, 4]. In 2019, an estimated 215 million malaria cases in the World Health Organization (WHO) Africa region, accounting for 94% of the global malaria cases and this contributed to 51% of malaria deaths globally [2].

Malaria accounts for 25 to 40% of all outpatient visits to health facilities in Uganda, and it is responsible for nearly half of all inpatient paediatric deaths [5, 6]. A number of environmental, climatic, seasonal, and ecological factors determine the occurrence and intensity of malaria transmission. For instance, while rain fall determines the availability of breeding habitats for mosquito vectors, temperature determines the length of mosquito larvae development and the rate of growth of the malaria parasites inside the vector [7–10].

For a long time, malaria diagnosis relied on traditional light microscopic examination of Romanowsky-stained blood smears. Also, the use of rapid diagnostic tests (RDT) has become a standard of care in resource-constrained settings [11, 12]. While they are less expensive and have a shorter turnaround time, they have a high error rate (false positives, false negatives, and species misidentification), particularly at low parasitaemia [12, 13]. Light microscopy has generally been considered the gold standard for malaria diagnosis due to its advantages, such as species identification and quantification, as well as its use of less sophisticated equipment [12–14]. Despite these benefits, the use of light microscopy as the gold standard has been paradoxical as it has a predicted detection limit of fifty to one hundred parasites per microlitre of blood [13]. Resultantly, microscopy is an imperfect reference standard, necessitating the evaluation of alternative methods sensitive enough to detect low levels of parasitaemia in asymptomatic infections [15], as well as the use of light microscopy to supplement or replace parasitological examination to reduce diagnostic errors [16]. Because anti-malarial treatments are dependent on the parasites that cause the disease, it is critical to track the distribution of *Plasmodium* species and decipher the anti-malarial drug pattern [17]. One approach has been to use molecular-based amplification of DNA. This method uses conventional or real-time quantitative PCRs (qPCR) [13, 18, 19]. The assays have high sensitivity and specificity, and can help detect parasites that would otherwise be missed in the peripheral blood circulation [13, 20]. For malaria species identification, 18S nested PCR^1^ has been widely used in identification using the conventional PCR assay. With real time high resolution melting (HRM) analysis, each species produces a diagnostic amplicon-specific melting profile [21, 22]. The quality of the DNA obtained from blood samples is important for effective PCR testing for *Plasmodium* species identification. Several molecular and epidemiological studies have used blood spots on filter paper and Giemsa-stained blood smears as a DNA source [22–24]. Long-term storage of DBS allows for retrospective studies to determine changes in infecting malaria species and the progression of drug resistance over time [25, 26]. Moreover, anti-malarial drug resistance^2^ is becoming a major issue, emerging as a result of parasite mutation rate, the overall parasite load, strength of the drugs selected, treatment compliance and poor adherence to malaria treatment guidelines, among other factors [17]. Resistance to chloroquine (CQ), sulfadoxine-pyrimethamine, and artemisinin-based combination therapy (ACT) has been reported in most parts of sub-Saharan Africa, but no new drugs have been developed, and most populations continue to use the artemether-lumefantrine as first-line therapy [27, 28]. Resistance to anti-malarial compounds is a major issue confronting malaria control programmes, and today, resistance to nearly all established anti-malarial compounds has been reported [28, 29]. Currently, no other anti-malarial treatment has the same efficacy and tolerability as ACT [30, 31].

The district malaria profiles in the Kigezi region revealed that Kisoro district had 37 confirmed malaria cases per 1000 population, with a 100% proportion of malaria confirmed cases, and 5.3 malaria deaths per 100,000 populations, with a 0.92% case fatality rate, compared to 191 confirmed malaria cases, a malaria mortality rate of 9 deaths per 100,000 populations, and a 5% case fatality rate nationally [19]. In addition, the report on the status of malaria epidemics in Uganda revealed malaria outbreaks in Nwoya and Kisoro districts, which were largely attributed to the migration of Sudanese and Congolese citizens fleeing insecurity in these countries; these observations were based on microscopy; to make informed epidemiological decisions, an accurate determination of malaria infection as well as a documentation of anti-malarial resistance markers are required. This study determined the different *Plasmodium* species and molecular identification of anti-malarial resistance markers of *Plasmodium falciparum* from patients attending Kisoro Hospital in Kisoro district, Southwestern Uganda.

## Methods

### Study design

This was a cross-sectional study in which samples were analysed to identify *Plasmodium* species and anti-malarial resistance markers of *P. falciparum* from patients with clinical symptoms of malaria at Kisoro District Hospital.

### Study area, population, sample size, and recruitment criteria

Kisoro Hospital in Kisoro Municipality, Bufumbira South Constituency, southwestern Uganda, was where the study was conducted. Kisoro District hospital serves as a referral centrefor the district as well as patients from the neighbouring Democratic Republic of the Congo (DRC) and Rwanda. The study conveniently considered 152 patient blood samples which tested positive on blood smear microscopic examination.

### Sample collection and analyses

Venous blood samples from patients who tested positive for malaria between March and August, 2020 and DBS was prepared on Whatmann® 903™ filter paper (Ref: 10530143 Rev.AA) by putting a drop of the blood sample from the finger prick into each cycle of the filter paper. The samples were air dried for up to 24 h away from wind and direct sunlight. After air drying, the filter papers were placed into ziplock bags with two sachets of desiccant in each pack and stored at room temperature (25 °C to 28 °C). The samples were later analysed at the Genomic and Translational Laboratory, Microbiology Department of Mbarara University of Science and Technology.

### Smear preparation, staining, and examination

After disinfection using 70% alcohol swab, blood was collected from the patients using a 2 ml ethylene di-amine tetra acetic acid vacutainer. A drop of blood was placed on a microscope slide and spread to cover an area of about 1 square centimetre (cm^2^) to create a thick blood smear for microscopy. The film was spread thin enough so that it appeared transparent. It was air-dried, and care was taken not to fix the thick smear, and subsequently stained with Field’s staining technique. The smear was air-dried and examined using high power magnification. Before a slide was declared negative for microscopy, a minimum of 200 microscopic fields were examined at a magnification of X1000 with oil immersion optics.

### Filter paper preparation

The remaining blood was spotted onto Whatmann® 903 filter paper cards (GE Healthcare, Life Sciences), as described previously.

### DNA extraction

This was carried out in accordance with the Zymo Research kit protocol (Zymo Research quick g-DNA miniPrep Kit, Cat #3025, 17062 Murphy Ave. Irvine, CA 92614, USA) [32]. Specifically, half of the blood spot was removed with a sterile surgical blade and placed in a 1.5 mL conical centrifuge/Eppendorf tube. The blood spot was then filled with 500 L of Zymo Lysis buffer, which was vortexed for about 15 s. For 10 min, the tube was allowed to incubate at room temperature. The entire contents of the tube (cell Lysate) was transferred to the Zymo-Spin column, which was placed in a filtrate collection tube. For one minute, the tube was spun in a micro-centrifuge at 10,000 rpm. The spin-column was removed and transferred to a new collection tube, and 200 mL of DNA Pre-wash buffer was added. The tube was spanned again for 1 min at 10,000 rpm. The Column was moved to a new collection tube, and 500 mL of g-DNA wash buffer was added. The content was centrifuged for 1 min. The Spin Column was transferred to a new 1.5 mL microcentrifuge tube, and 50 mL of DNA elution buffer was added. The tube was allowed to stand at room temperature for 5 min before being centrifuged at 15000 rpm for 30 s and the DNA was collected in the 1.5 mL Eppendorf tube. DNA was kept at −20 degrees Celsius until it was used.

### HRM plasmid and parasite DNA controls

*Plasmodium* species plasmid controls were obtained from the American Type Culture Collection (ATCC) (Manassas, VA, USA) and used as controls for the assays performed. The plasmids included *Plasmodium falciparum* (MRA-177-Pf small subunit [SSU] rRNA nest 1 PCR plasmid clone 8; lot 5946054), *Plasmodium malariae* (MRA-179Pm SSU rRNA nest 1 PCR plasmid clone 34; lot 61909614), *Plasmodium ovale* (MRA-180-Po SSU rRNA nest 1 PCR plasmid clone 54; lot 59467055), and *Plasmodium vivax* (MRA-178-Pv SSU rRNA nest 1 PCR plasmid clone 16; lot 58067149) and *P. falciparum* laboratory clone (strain Dd2, MRA-331).

### PCR cycling and HRM

2× HRM master mix (Type-It HRM PCR kit; Qiagen Benelux) and primers targeting the *Plasmodium* DNA, i.e., PL1473 F18 (5’-TAA CGA ACG AGA TCT TAA-3’) and PL1679 R18 (5’-GTT CCT CTA AGA AGC TTT-3’) were used [33]. These primers hybridize to 18S rRNA gene regions that are conserved across human *Plasmodium* species and surround variable regions, allowing species differentiation during HRM analysis. On a cooled sample rack, reagents were prepared, and each 25uL PCR mixture contained 12.5 of Type-It HRM PCR master mix and 0.7 M (final concentrations) of forward and reverse primers (PL147359 F2 and PL1706 R2, respectively), 3 uL of template DNA, and 6uL of RNase-free water in the final reaction mixture. The following conditions were used for PCR cycling: 95 °C for 5 min, followed by 40 cycles of 95 °C for 10 s, 57 °C for 30 s, and 72 °C for 10 s. The ramp ranged from 65 °C to 95 °C, increasing 0.5 °C in each step, to perform an HRM analysis on the resulting PCR product. The steps of thermocycling, fluorescent detection, and HRM were carried out in a BIORAD CFX 96 C1000 Touch real-time PCR^3^ thermocycler with a 0.2 uL PCR reaction tube.

### Plasmodium species determination

For analysis, the CFX 96 Manager software (version 3.1.1517.0823, BIO-RAD) was used. The software plotted the negative of the change in a fluorescence versus temperature d(RFU)/dT) for HRM analysis. Within this -dRFU/T plot, each *Plasmodium* species produced a distinct thermal profile as well as a peak that allowed species differentiation. Based on control samples, a manual fluorescence threshold was established; only fluorescence data above this threshold were considered. The species-specific peaks are used to assign species to the tested samples for automatic calling.

### Anti-malarial drug sensitivity markers detection

To detect *pfcrt* (*pfcrt* 76 T) and *kelch* 13 (C580Y) mutations, quantitative PCR was used followed by High-Resolution Melt (HRM) analysis. The prevalence of the *pfcrt* sensitive wild type (CVMNK) and resistant *pfcrt* resistant haplotypes (CVIET and SVMNT) were determined using the CFX 96 C1000 touch Real PCR System. Pail resistant isolate and Dd2 sensitive strains were used as *kelch13* controls.

### Sensitivity of HRM assay for *pfcrt* in single and mixed strains reactions

The method's ability to detect a low amount of haplotype in a mixture of wild-type and mutant-type *pfcrt* alleles was also tested. The DNA concentrations of the reference strain MRA102G (CVMNK) and MRA-150G (CVIET) were adjusted to 1.0 ng/l and combined to yield the following ratios: CVMNK/CVIET ratios of 60/40, 20/80, and 80/20 and used to assess sensitivity in mixed haplotypes.

### Primers

Two different primers and probe were used to search for anti-malarial drug resistance mutations in the Plasmodium *falciparum* transporter gene (pfcrt) for chloroquine resistance and another primer pair and probe for ACT (AL, Coartem®) (*kelch13*). This study focused only on the *kelch13* C580Y mutation for resistance to the ACTs. The primers included the following; for PfCRT: 5’GTAAAACGACGGCCAGTTTCTTGTCTTGGTAAATGTGCTCA-3’, Reverse primer: 3’CAGGAAACAGCTATGACCGGATGTTACAAAACTATAGTTACCAAT-5’, HRM Probe: 5’-GTGTATGTGTAATGAATAAAATTTTTG (3SpC3)-3’. The HRM probe, which was unlabelled, detected mutations in the 72–76 codon region. The C3 spacer on the probe's end was required to prevent the probe from extending during PCR amplification. The probes disassociated from the mutant and wild-type template DNA during the HRM steps at different melting temperatures. For *kelch13* propeller gene, the following primers and probe were used: Forward Primer: 5’-GGCACCTTTGAATACCC-3’ Reverse Primer: 5’-CATTAGTTCCACCAATGACA-3’, Probe: AGCTATGTGTATTGCTTTTGAT-BLOCK (C3 spacer) (Daniels, Volkman and Wirth, pers. commun.), *kelch13 controls*: Genomic DNA controls used: MRA-152G (7G8 gDNA-double mutant haplotype SVMNT), MRA-155G (HB3 gDNA-wild type haplotype CVMNK), MRA-156G/150G (Dd2 gDNA-triple mutant haplotype CVIET), and MRA-175G (7C424-triple mutant haplotype CVIET). For *kelch13* investigation, genomic controls used were: MRA-1236 (PAIL resistant- C580Y allele [34], (Dd2 sensitive-wild type).

### Setting up the PCR

*Primer dilution* all primers were initially re-suspended at a concentration of 100 µM and then diluted to working stocks of 10 µM*.*

*Reaction/Master mix* the PCR mixture was pipetted into 0.2 ml PCR tubes, samples added into each tube and the tubes loaded into the CFX 96 Real-time PCR platform, where the assays were run under the cycling conditions listed below:

*Pfcrt assay cycling conditions* the following PCR reaction conditions were used: 95 °C for 6 min; 45 cycles of 95 °C for 15 s and 55 °C for 1 min; followed by 94 °C for 30 s and 25 °C for 30 s for heteroduplex formation; and 15 °C for storage. Melt curve analysis was performed on the CFX 96 C1000 touch real-time PCR Cycler (BIORAD).

*Kelch 13 assay cycling conditions (95 °C for 15 min)* 45 cycles of 95 °C for 30 s, 56 °C for 30 s, 72 °C for 30 s, 98 °C for 2 min, 40 °C for 2 min (facilitated heteroduplex formation, melting program completed for 90 s at initial temperature), melting at 0.2 °C/s from 65 °C to 95 °C (minimum dwell time of 2 s) [35].

### Quality control

Slides were routinely read by two independent, well-trained laboratory personnel. A third slide reading was used to evaluate discordant results, and the final result diagnosis was based on the majority agreement of experienced laboratory personnel. Genomic control DNA was used in all the PCR assays performed. Curves and melting temperatures between the genomic control DNA and the clinical samples were compared. Those that matched the control profiles were considered as being species or having the same alleles as the controls and were therefore, called accordingly.

### Data analysis

The information was entered into a Microsoft Excel spreadsheet. *Plasmodium* species were identified by comparing the control and test sample HRM-PCR derivative curves, a table was used to summarize the distribution of the various *Plasmodium* species, and the anti-malarial drug profile was presented using the HRM-PCR derivative curve.

### Ethical consideration

The study obtained ethical approval from Mbarara University of Science and Technology Research and Ethics Committee (REC). Administrative permission was obtained from the Hospital Medical Superintendent, Kisoro District Hospital. Consent/assent was obtained from each study participant. To ensure the anonymity of the research participants, study codes were used throughout the study.

## Results

### Study profile

See Fig. 1.

**Fig. 1:**
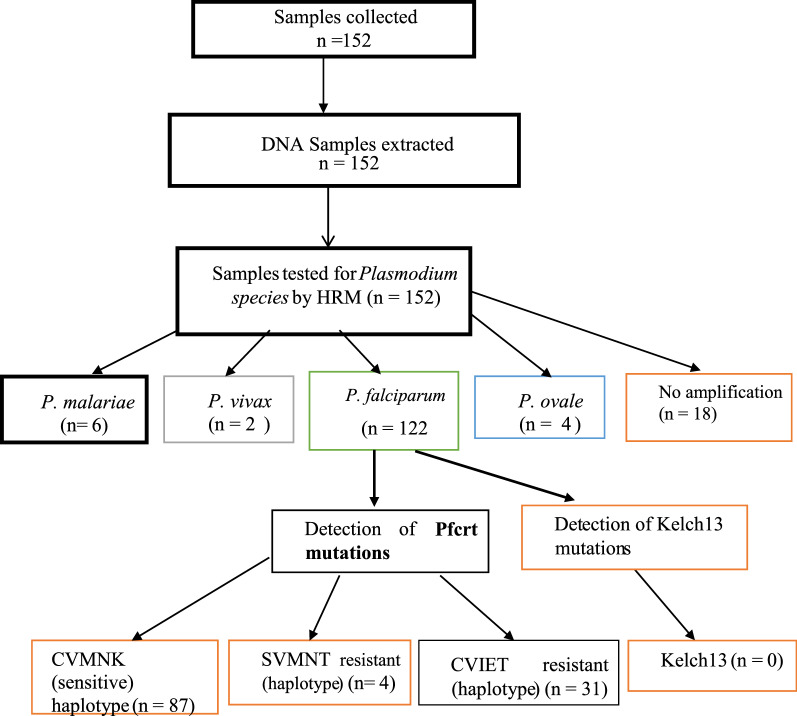
Study flow and results obtained

### Socio-demographic profile of the study participants

Of the 152 participants, the majority were females (N = 98, 64.5%). Participants mean age was 34.9 years (range: 2 months–81 years), as presented in Table 1.

**Table 1:** Demographic profiles of study participants

Variable	Frequency (n)	Percentage (%)
Age category (Years)		
14 and below	51	33.6
15–44	67	44.1
45 and above	34	22.4
Gender		
Male	54	35.5
Female	98	64.5
Country of origin		
Uganda	138	90.8
Rwanda	05	3.2
Burundi	00	0
Democratic Republic of Congo	09	5.9

### Plasmodium species identification results

A total of 152 samples were collected. Of these, 18 samples did not amplify and these were regarded as negative by PCR-HRM. Of the 134 that showed amplification, all the four *Plasmodium* species were detected and *P. falciparum* was the most prevalent species (N = 122, 91.04%), while other non-falciparum species, such as *Plasmodium malariae* (N = 6, 4.48%), *Plasmodium ovale* (N = 4, 2.99%) and *Plasmodium vivax* (N = 2, 1.49%) were also detected. The distribution of different *Plasmodium* species is shown in Table 2.

**Table 2:** Proportion of the *Plasmodium species* detected

*Plasmodium species*	n (%)
*Plasmodium malariae*	6 (4.48)
*Plasmodium vivax*	2 (1.49)
*Plasmodium ovale*	4 (2.99)
*Plasmodium falciparum*	122 (91.04)

### Resistance markers

Of the 122 samples, 87 (71.3%) showed a sensitivity to chloroquine representing the wildtype haplotypes (CVMNK), 4 (3.3%) were shown to be the double mutant resistant haplotype (SVMNT), and the rest of the samples, 31 (25.4%) were shown to be the triple mutant resistant haplotypes (CVIET), as summarized in Table 3.

**Table 3:** Summary of anti-malarial drug resistant and sensitive haplotypes

Haplotype	Phenotype	n (%)
CVIET	Triple mutant	31 (25.4)
SVMNT	Double mutant	4 (3.3)
CVMNK	Wild type	87 (71.3)

### K13 results

**Fig. 2:**
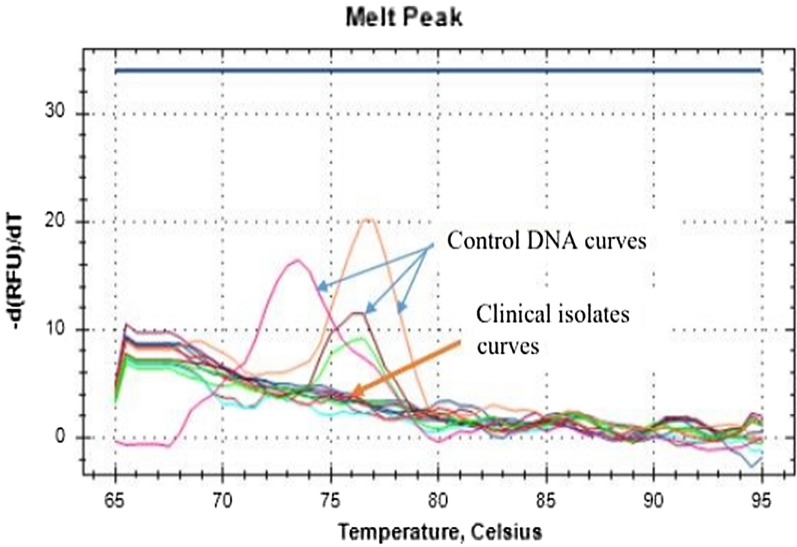
HRM run of *Kelch 13* genomic DNA controls and representative clinical samples

Only 580Y mutation was analysed. From the *P. falciparum* isolates tested, none of them showed any positivity to *kelch13* mutation (Fig. 2).

## Discussion

A total of 152 patient samples were found to be positive by smear microscopy, but 18 of them (11.84%) tested negative by HRM-PCR. The difference in positive detection between the two methods could be attributed to some DNA that could have been lost during DBS preparation, processing, and storage. Previous investigations suggested a reduction in the quantity and quality of the resultant DNA as a result of DBS storage, which has serious consequences because high ambient temperatures have a detrimental impact on the quality of DNA [25, 36].

A total of 134 samples showed amplification, confirming that they were *Plasmodium* species. The investigation revealed the presence of all four *Plasmodium* species (*P. falciparum*, *P. malariae*, *P. ovale*, and *P. vivax*) in the community, demonstrating the malarial endemicity. This is in agreement with a similar study carried out in Kampala, Uganda [7].

*Plasmodium falciparum* accounted for 91.04%, as expected, and this pattern is consistent with earlier research [37–40]. For example, 87.3% of *P. falciparum* infections were recorded in a Ghanaian study [41]. Similarly, *P. falciparum* was found in 92% of the infections diagnosed with HRM-PCR in a study employing stored blood slides and pellets [22]. *Plasmodium falciparum* was found in 97% of all malaria infections in Zambia's southern and western districts [35]. In contrast, research conducted in Brazaville, Republic of Congo, discovered a 100% prevalence of *P. falciparum* among positive patients [42]. This contradicts the findings of the current study. In general, these findings show that *P. falciparum* is extremely common and is the leading cause of malaria in this study setting [43]. It is well known that *P. falciparum* is associated with significant morbidity and death due to its severe clinical manifestations, which reflects the poor results in our situation [44, 45]. Non-falciparum species were also found; *P. malariae* accounted for 4.48% (N = 6), *P. ovale* was found in 2.99% (N = 4), and *P. vivax* was found in 1.49% (N = 2). This pattern is consistent with earlier reports [46, 47]. Non-falciparum species were also detected, which is consistent with findings of other studies [22]. This could be attributed to environmental weather changes as well as unsafe human immigration practices as a result of the inflow of tourists and refugees into the country [48]. As a result, the existence of these non-falciparum *Plasmodium* species is not unusual. This pattern of infection with non-falciparum species reflects the complexities of malaria case care in our setting [35, 41]. Non falciparum species if mistaken for falciparum may lead to wrong treatment which ends up in chronic infection/recurrent infection [46]. Non falciparum species are distinguished by chronic infections (*P. malariae*) or dormant lifecycle stages (*P. vivax* and *P. ovale*), and as a result of their chronicity and the presence of hypnozoite stages, they account for an increasing proportion of infections [49, 50]. Furthermore, non-falciparum species should support the urgent need for determining the infecting *Plasmodium* species, which is important in terms of treatment because specific *Plasmodium* infections can cause rapidly progressive severe illness or death, whereas other *Plasmodium* infections are less likely to cause severe manifestations or have different drug resistance patterns [39, 50]. The findings of this study revealed that 87 (71.3%) parasites were carrying wild-type strains (CVMNK haplotypes) sensitive to chloroquine. The increasing prevalence of the *pfcrt* wild type haplotype (CVMNK) is also linked to the use of artemether-lumefantrine in Uganda, lumefantrine is selecting in opposite direction to chloroquine. The remaining parasites tested had resistant *pfcrt* haplotypes; CVIET (triple) and SVMNT (double) mutant strains, both of which are linked to chloroquine resistance. Treatment with chloroquine and amodiaquine selects for these same mutations. The discovery of *P. falciparum* chloroquine sensitive haplotypes is consistent with previous research findings in Tororo, Uganda [51] and other countries in the region [52–54]. A study conducted on Hainan Island, China, for example, discovered a high number of sensitive CVMNK haplotypes [55]. Similarly, a meta-analysis for sub-Saharan Africa revealed a large number of sensitive strains (CVMNK) [56]. Chloroquine has not been widely used for malaria treatment in the past decade, nevertheless, a 28.7% combined resistance to chloroquine (CVIET & SVMNT haplotypes) was detected. This resistance pattern indicates that the chloroquine-resistant allele is likely to revert to the sensitive allele in the population [57–60]. In our study, only the C580Y mutation for K13 was analysed and none of the *P. falciparum* samples had a C580Y *kelch13* mutation. Although some of the parasites had chloroquine resistance, they were all sensitive to artemisinin-based combinations, which is reassuring. This supports previous findings and provides the much-needed hope that ACT remains an effective therapy in the study setting [59–61].

## Conclusion

This study found a high prevalence of pan *Plasmodium* species in the community served by Kisoro Hospital, confirming the previously reported rising malaria burden in this study setting. Furthermore, non-falciparum species were also detected by PCR, there is concern that these species may continue to drive transmission but go undetected, as RDTs used in most government facilities in the study setting detects only *P. falciparum*. While this study confirms that *P. falciparum* predominates, a significant proportion of these non-falciparum infections, 8.96%, do occur in this region as well. According to the findings of this study, it is critical to expanding surveillance activities by increasing capacity to diagnose and detect non-falciparum species using Pf/Pan RDTs as well as molecular techniques like HRM PCR analysis used in this study to monitor non-falciparum infections.

The following limitation should be considered when interpreting the findings of this study:

The study looked at chloroquine and C580Y mutation for artemisinin anti-malarial drug targets only. Mutations in other anti-malarial agents in use such as prevalence of *pfdhps* and *pfdhr* were not studied. Furthermore, in this study, a phylogenetic analysis of the parasite population was not conducted, which could have aided in dissecting this relationship by defining parasite population diversity and the relatedness.

## Data Availability

We did not obtain consent to share data obtained, however, the datasets used and/or analysed during the current study are available from the corresponding author (godfreymanira@gmail.com) on request.

## Abbreviations

DBS: Dried Blood Spot
DNA: Deoxyribonucleic Acid
HRM: High-Resolution Melting
PCR: Polymerase Chain Reaction
*PfCRT*: *Plasmodium falciparum* Chloroquine Resistance Transporter
*pfcrt* CMVNK: Cysteine-Valine-Methionine-Asparagine-Lysin
*pfcrt*SVMNT: Serine-Valine-Methionine-Asparagine-Threonine
*pfcrt*CVIET: Cysteine-Valine-Isoleucine-Glutamate-Threonine

## Footnotes

1 A PCR modification designed to reduce non-specific binding in products caused by amplification of unexpected primer binding sites.

2 The ability of Plasmodium species to survive after drug absorption at concentrations greater than the patient's tolerance

3 This is a molecular biology laboratory technique that uses PCR to monitor the amplification of a specific deoxyribonucleic acid (DNA) molecule during the PCR
